# Supplementary material for: Antibacterial Gelatin Composite Hydrogels Comprised of In Situ Formed Zinc Oxide Nanoparticles
Source: Polymers (Basel). 2023 Oct 3;15(19):3978. doi: 10.3390/polym15193978 (PMC10575203; doi:10.3390/polym15193978)
Supplement: Supplementary file 1 [file polymers-15-03978-s001.zip › polymers-2634194-supplementary.pdf]

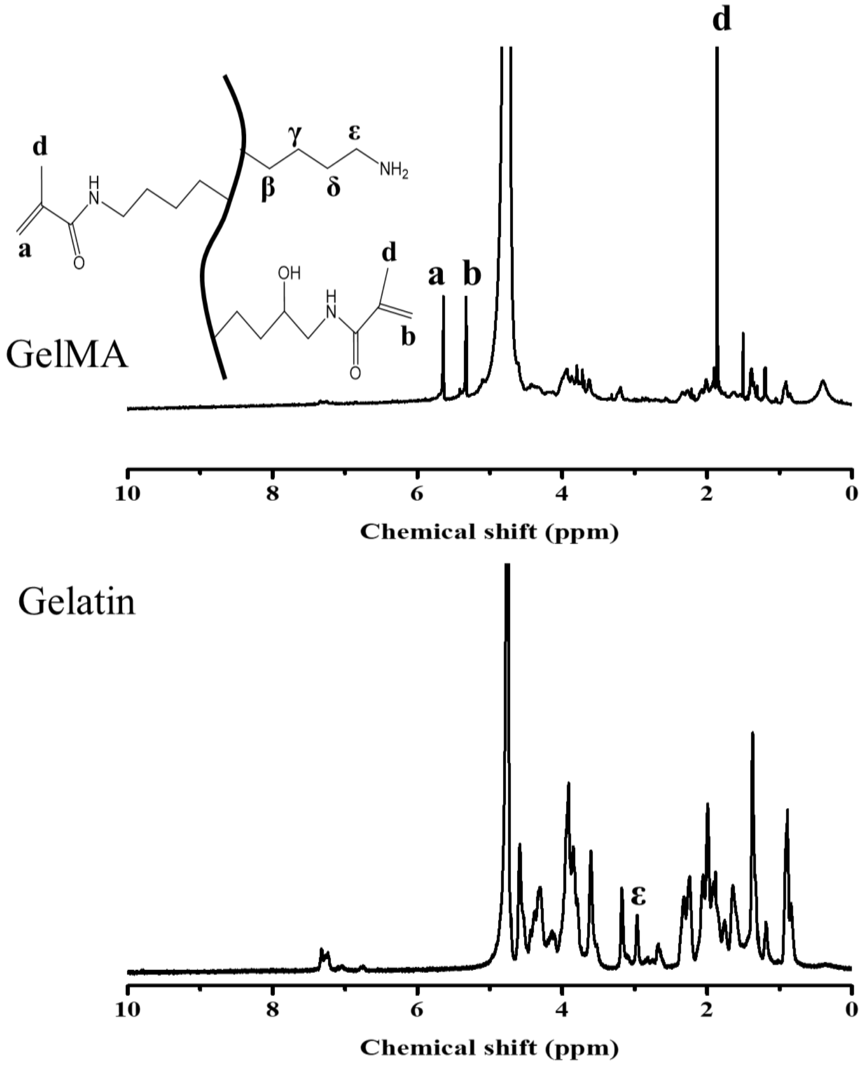

Figure S1. ^{1}H NMR spectra of GelMA and gelatin dissolved in D_2O.

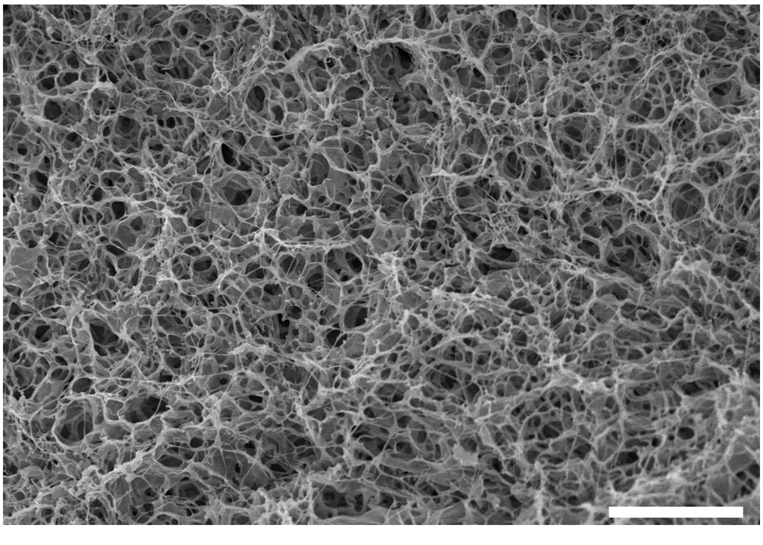

Figure S2. Scanning electron microscopy section images of cross section of Gelatin hydrogel network. The scale bar was 30 μm.

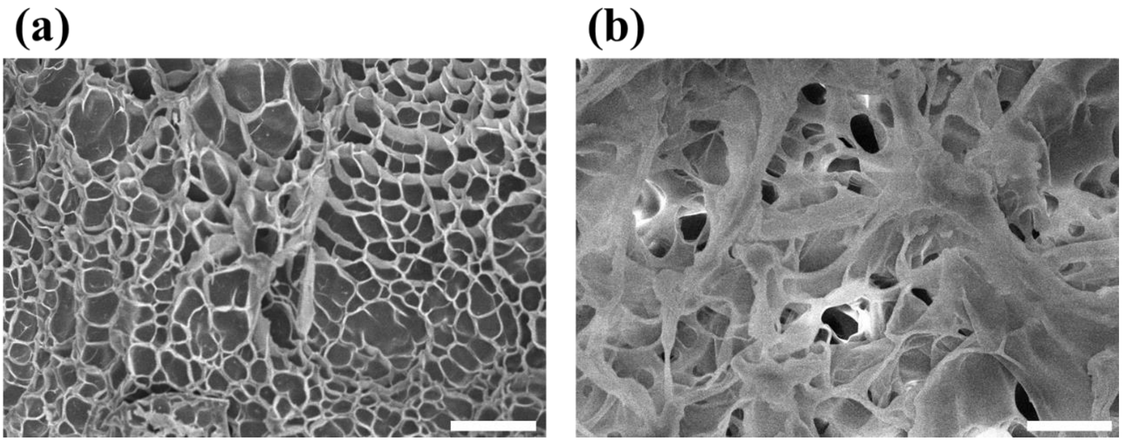

Figure S3. Scanning electron microscopy section images of cross section of (a) Gelatin/ZnO1 hydrogel network; the scale bar was 100 μm, and (b) Gelatin/ZnO10 hydrogel network; the scale bar was 30 μm.

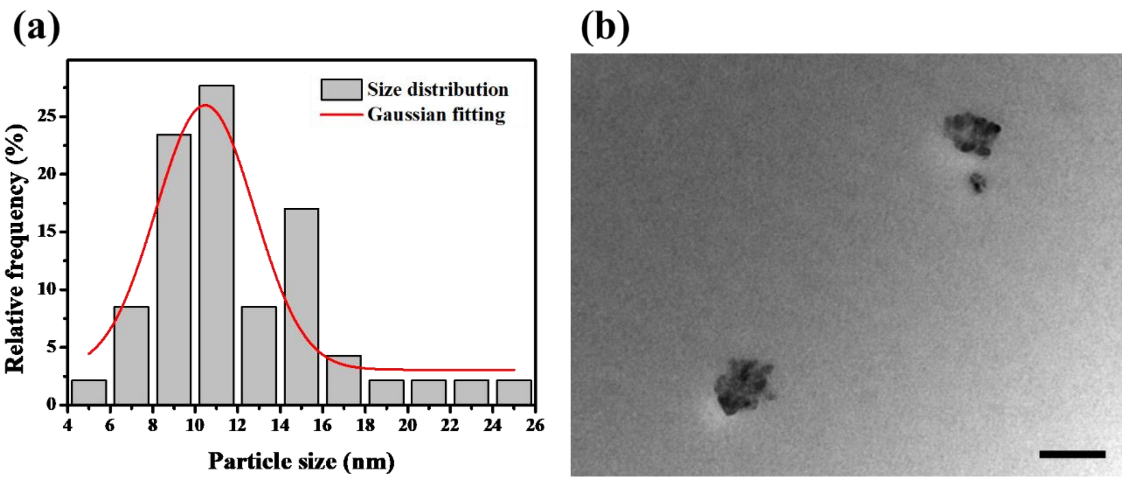

Figure S4. (a) Particle size distribution and Gaussian fitting (n = 50), and (b) TEM image of Gelatin/ZnO1 prepared at 20 mg mL^{-1} in DI water with 3 mg bromelain. The scale bar was 100 nm.

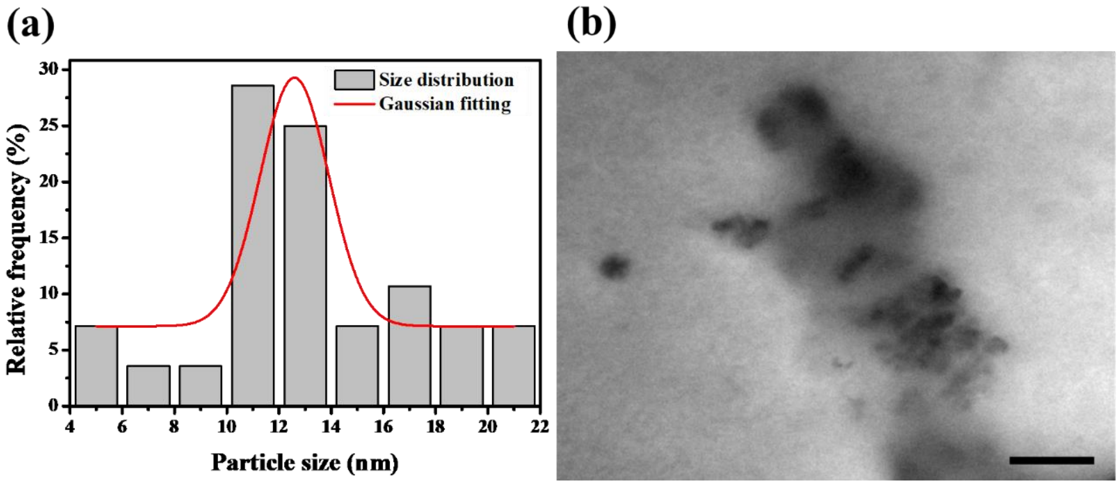

Figure S5. (a) Particle size distribution and Gaussian fitting (n = 30), and (b) TEM image of Gelatin/ZnO10 prepared at 20 mg mL^{-1} in DI water with 3 mg bromelain. The scale bar was 100 nm.

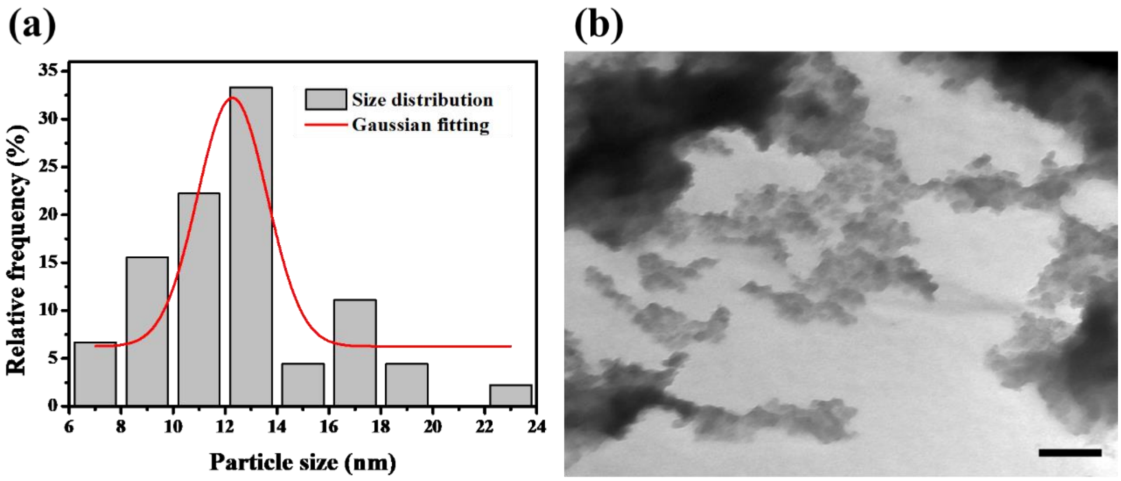

Figure S6. (a) Particle size distribution and Gaussian fitting (n = 50), and (b) TEM image of Gelatin/ZnO30 prepared at 20 mg mL^{-1} in DI water with 3 mg bromelain. The scale bar was 100 nm.

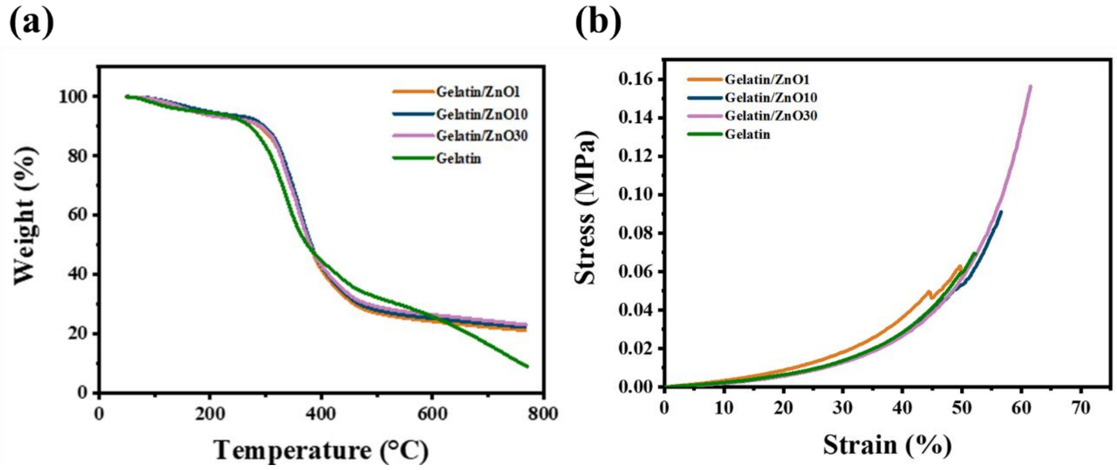

Figure S7. (a) Thermogravimetric analysis of freeze-dried gelatin hydrogel and ZnO-loaded gelatin hydrogels and (b) stress-strain curve of gelatin and ZnO-loaded gelatin hydrogels.

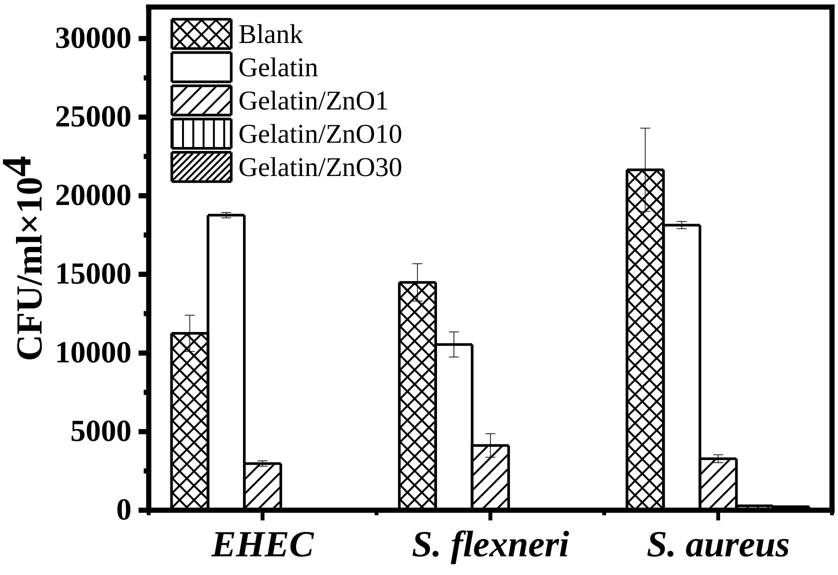

Figure S8. The antibacterial activity of *EHEC*, *S. flexneri*, *S. aureus* treated with Gelatin hydrogel and ZnO-loaded hydrogels for two hours (n = 3).
